# 3W Dataset 2.0.0: a realistic and public dataset with rare undesirable real events in oil wells

**DOI:** 10.1038/s41597-026-07225-z

**Published:** 2026-04-24

**Authors:** Ricardo Emanuel Vaz Vargas, Afrânio José de Melo Junior, Claudio Benevenuto de Campos Lima, Jean Carlos Dias de Araújo, Mateus de Araujo Fernandes, Rogério Leite Alves Pinto, Jader Riso Barbosa, Celso José Munaro, Patrick Marques Ciarelli, Flávio Miguel Varejão, Felipe Muntzberg Barrocas, João Neuenschwander Escosteguy Carneiro, Matheus Lima Scramignon, Rodrigo Castello Branco, Tiago Pereira Siqueira, Eduardo Toledo de Lima Junior, Igor de Melo Nery Oliveira, Lucas Gouveia Omena Lopes, Lucas Pereira de Gouveia, Guilherme Fidelis Peixer, Jaime Andrés Lozano Cadena

**Affiliations:** 1 Petróleo Brasileiro S.A. (Petrobras), Rio de Janeiro, Brazil; 2 Craft & Hawkins Department of Petroleum Engineering, Louisiana State University, Baton Rouge, United States of America; 3 Department of Electrical Engineering, Federal University of Espírito Santo (UFES), Vitória, Brazil; 4 Department of Informatics, Federal University of Espírito Santo (UFES), Vitória, Brazil; 5 HybridAI, Rio de Janeiro, Brazil; 6 Infotec Brasil, Rio de Janeiro, Brazil; 7 Laboratory of Scientific Computing and Visualization (LCCV), Center of Technology (CTEC), Federal University of Alagoas (UFAL), Maceió, Brazil; 8 Research Laboratories for Emerging Technologies in Cooling and Thermophysics (POLO), Department of Mechanical Engineering, Federal University of Santa Catarina (UFSC), Florianópolis, Brazil

**Keywords:** Engineering, Computer science

## Abstract

In the oil industry, undesirable events in oil wells can cause economic losses, environmental accidents, and human casualties. In 2019, recognizing the importance and the lack of public datasets related to undesirable events in oil wells, Petrobras developed and publicly released the first version of the 3W Dataset, which is essentially a set of Multivariate Time Series labeled by experts. Since then, the 3W Dataset has been developed collaboratively and has become a foundational reference for numerous works in the field. This data article describes the current publicly available version of the 3W Dataset, which contains additional instances, more variables, and a new label. Furthermore, a new data structure has been developed to make data access more robust and efficient. The detailed description we provide encourages and supports the 3W community and new 3W users to improve previous published results and to develop new robust methodologies, digital products and services capable of detecting undesirable events in oil wells with enough anticipation to enable corrective or mitigating actions.

## Background & Summary

Undesirable events cause different types of damage to the oil industry, including economic losses, environmental accidents, and human casualties^1^.

Abnormal Event Management (AEM) refers to systematic detection, diagnosis, and mitigation of unexpected or irregular events within complex industrial systems^2^. In the oil industry, where operational safety, environmental protection, and economic performance are tightly coupled, AEM is crucial for minimizing the impact of undesirable events. The integration of Artificial Intelligence (AI) and Machine Learning (ML) based solutions into AEM have shown promise for Early Detection^3^ of undesirable events across different industries^4^. By analyzing large streams of operational data, such as pressure, temperature, vibration, and flow rates, algorithms can identify subtle patterns that precede abnormal conditions, allowing early intervention and preventive maintenance strategies^5^. An essential requirement for the success of this type of approach is the use of high-quality datasets^6,7^.

This need was recognized by Petrobras — the largest oil company in Brazil, operating in the exploration, production, refining, marketing, and transportation of oil, natural gas, and energy^8^ — which developed the 3W Dataset and published its first version in 2019, as described in detail by Vargas *et al*.^9^.

The 3W Dataset is a collection of Multivariate Time Series (MTS)^10,11^, referred to as instances, which have been labeled by experts from Petrobras and its partners. The name ***3W*** was chosen because this dataset comprises instances derived from ***3*** different sources (real, simulated, and hand-drawn) which contain undesirable events that occur in oil ***W***ells. Each instance may either represent 100% of the data associated with normal operating conditions or contain data partially related to a specific type of undesirable event. The core idea behind this dataset is to enable the learning (modeling) of temporal patterns or signatures across multiple variables, distinguishing between normal conditions and various types of undesirable events.

The main features of the 3W Dataset are as follows. Its real instances correspond to data collected directly from real industrial environments. Characteristics such as frozen variables, missing variables, and outliers are intentionally left untreated. This approach aims to encourage and enable the development of methodologies and digital products capable of dealing with real-world challenges. By preserving these typical characteristics, a high-quality and useful dataset is generated^12^. Simulated instances have been added because some types of undesirable events are rare in real-world operations. Hand-drawn instances have been added to address events that are not only rare but also challenging to simulate. Experts can use their understanding of variable behaviors during such events to manually create these instances to mirror real-world conditions as closely as possible.

Due to its features, the 3W Dataset can also serve as a foundational resource for training basic models in Transfer Learning frameworks^13^. These frameworks rely on a large and generalized dataset as a reference or starting point for training models to address different but related challenges, particularly when an adequate training dataset for the target task is unavailable. This approach is widely used in Deep Learning^14^, especially in scenarios requiring large amounts of training data.

The 3W Dataset is managed using Semantic Versioning^15^, with its initial release designated as version 1.0.0.

Since its initial release, the 3W Dataset has been explored by several people who make up the 3W Community^16^, including independent professionals and representatives of research institutions, startups, companies, and oil operators from different countries.

The 3W Community has contributed to the development and publication of numerous works, forming a substantial scientific framework focused on the Early Detection of undesirable events in oil wells. This framework is composed of books, conference papers, data articles, doctoral theses, final graduation projects, journal articles, master’s degree dissertations, repository articles, and specialization monographs. Publications identified so far that cite the 3W Dataset are listed in the 3W Project repository^17^.

In 2022, Petrobras officially launched the 3W Project as the inaugural pilot of the Open Lab Module within the Connections for Innovation Program^18^. The purpose of this module is to encourage open and collaborative project development on the Internet, particularly through the GitHub platform^19^. Since then, the 3W Dataset has been maintained and developed in its dedicated corporate Git repository^17^ on GitHub.

As part of the 3W Project, the same Git repository also hosts the 3W Toolkit, a software package developed in Python 3^20^. The purpose of this toolkit is to facilitate and encourage the exploration of the 3W Dataset, as well as the development and comparison of different methodological approaches.

In addition to its two main resources — the 3W Dataset and the 3W Toolkit — the 3W Project repository also provides:

- A detailed description of motivation, strategy, ambition, governance, and other aspects of the 3W Project;
- A list of at least 145 published works that cite the 3W Dataset;
- Specifications of priority challenges (benchmarks);
- The 3W Project contributing guide;
- Information about the 3W Community;
- Overviews of the 3W Dataset developed by the 3W Community (demos);
- The 3W Community code of conduct;
- Release notes for the published versions of the 3W Dataset.

Since 2022, the 3W Dataset has been evolved under Petrobras’ leadership, and its current publicly available version designated as 2.0.0.

This data article describes the 3W Dataset 2.0.0^21^ and summarizes the advancements incorporated since version 1.0.0. The detailed description is intended to encourage and support the established 3W Community and also new users to improve previously published results and develop innovative methodologies, digital products, and services. These advancements aim to enable Early Detection of undesirable events in oil wells, allowing for timely corrective or mitigating actions.

## Methods

In summary, the 3W Dataset 2.0.0^21^ is composed of three types of instances generated by three methods, one per type of instance. These methods are described in this section and are based on the mathematical definition of MTS presented in the following subsection. According to this definition and to support both the 3W Project and this article, a nomenclature was developed and is detailed in the subsequent subsection. The types of instances and the methods associated with them are described in their own subsections. Some characteristics are common to the three methods and are, therefore, detailed in a separate subsection.

### Mathematical definition of multivariate time series

The chosen definition for MTS is the same as that used in the article that published the 3W Dataset 1.0.0^9^. This definition is reproduced below.

A dataset ***DS*** is a set of *m* MTS (S^*i*^ | *i* = {1, 2, …, *m*}, ∀ *m* ∈ Z, and *m* > 1) and is defined as ***DS*** = {***S***^1^, ***S***^2^, …, ***S***^*m*^}. Each MTS *i* is an instance composed of a set of *n* univariate time series (x^*i*^_*j*_ | *j* = {1, 2, …, *n*}, ∀ *n* ∈ Z, and *n* > 1) (also referenced as process variable or just variable), and is defined as ***S***^*i*^ = {*x*^*i*^_1_, *x*^*i*^_2_, …, *x*^*i*^_*n*_}. Each variable *j* that composes an MTS *i* is an ordered temporal sequence of *p*_*i*_ observations taken at the time *t* (*x*^*i*^_*j,t*_ | *t* = {1, 2, …, *p*_*i*_}, ∀ *p*_*i*_ ∈ Z, and *p*_*i*_ > 1). Therefore, each MTS *i* is considered in this work as a matrix defined as S^*i*^ = {*x*^*i*^_1,1_, *x*^*i*^_2,1_,…, *x*^*i*^_*n*,1_; *x*^*i*^_1,2_, *x*^*i*^_2,2_,…, *x*^*i*^_*n*,2_; *x*^*i*^_1,*pi*_, *x*^*i*^_2,*pi*_,…, *x*^*i*^_*n*, *pi*_}.

Note that all instances have a fixed number of variables *n*; all instances are composed of the same *n* variables; each instance can be composed of any number of observations *p*_*i*_; all *n* variables of an instance *i* have a fixed number of observations *p*_*i*_; and different instances can be composed of different numbers of observations.

### Nomenclature

The nomenclature used in this work is derived from the definition of MTS presented in the previous subsection. The specific terms and their descriptions are presented in Table 1.

**Table 1:** Terms that make up the nomenclature used in this work.

Term	Meaning
Variable	A physical quantity at a specific point in the production system of a particular well, from which measurements are acquired to produce a univariate time series *j*: *x*_*j*_
Timestamp	Instant *t* (date + time) at which values are acquired or generated and then associated with variables: YYYY-MM-DD HH:MM:SS
Observation	Vector with values from *n* variables of a single instance *i* acquired at a timestamp *t*: {*x*^*i*^_1,t_, *x*^*i*^_2,t_,…, *x*^*i*^_*n*,t_}
Label	Annotation provided by an expert regarding the well condition in terms of a particular property. The 3W Dataset 2.0.0^21^ includes two types of labels which are defined below: class label and state label. The labeling process is explained in Common Characteristics Among the Methods Subsection
Class label	Annotation provided by an expert regarding the well condition in terms of normality or the occurrence of an undesirable event. Class labels reveal whether each instance consists solely of data relating to normality or to a specific type of event. See additional explanation in Common Characteristics Among the Methods Subsection
State label	Annotation provided by an expert regarding the well condition in terms of operational status. State labels are related to the operational status of the respective well. See additional explanation in Common Characteristics Among the Methods Subsection
Sample	Part of an MTS, including all *n* variables and all observations between two timestamps
Period	A sample that respects the following two conditions: all its observations are labeled with the same class label, and it is not contained in another temporally larger sample with all observations labeled with the same class label. In other words, a period is the largest possible sample whose observations are labeled with the same class label
Instance	Collection of temporally sequential periods associated with a specific well
Type of event	Operational state in which a well can be found, including normality, failures, and undesired states
Dataset	Set of instances with multiple types of events

### Types of instances

As mentioned at the beginning of this section, the 3W Dataset 2.0.0^21^ is composed of three types of instances, denoted as real, simulated, and hand-drawn.

Each type of instance has been defined based on the origin of its data. Real instances were sourced from different Petrobras’ Plant Information Management Systems (PIMS)^22^, more precisely different AVEVA PI System^23^ environments. Data from simulated instances were generated with OLGA^24^, a dynamic multiphase flow simulator widely adopted by oil companies worldwide. Data from hand-drawn instances were created by Petrobras experts using a digital tool developed exclusively for this purpose.

The characterization of the 3W Dataset as realistic is primarily based on the real well data it contains. The inclusion of simulated and hand-drawn instances addresses the scarcity of examples for certain rare well events. As an effort to keep these instances as realistic as possible, they were designed with a strong emphasis on fidelity by relying on the knowledge of domain specialists.

Each type of instance, and therefore each data source, required the development of its own method for acquiring and labeling data. The common characteristics among the three developed methods are described in the following subsection. The particularities of each method are detailed in the subsequent subsections.

### Common characteristics among the methods

All instances, regardless of their type, are related to satellite-type offshore oil-producing wells that operate without manifold^25^. This is one of the most representative configurations for oil wells in many of the offshore production systems operated by Petrobras. These wells can alternate between different lifting methods^26^ over time, utilizing either the natural method or an artificial lifting method. The natural method is employed when the reservoir pressure is sufficient to produce hydrocarbons at a commercially viable rate without the need for additional energy. When reservoir pressure is insufficient, an artificial lifting method is required to introduce extra energy into the system and maintain production.

**Fig. 1:**
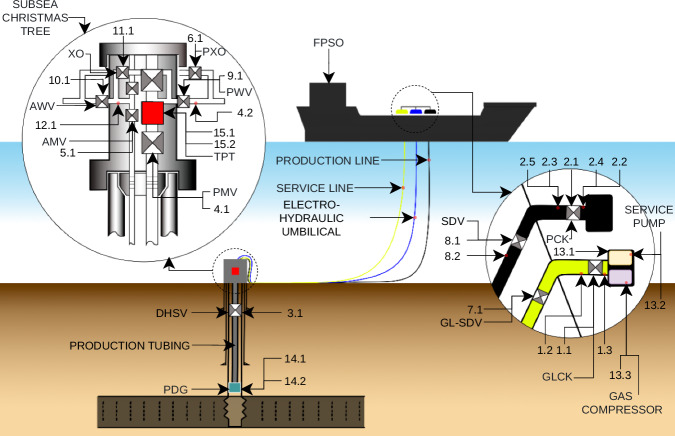
Diagram representing the considered scenario when designing the 3W Dataset 2.0.0^21^.

Figure 1 presents a diagram illustrating the scenario considered during the design of the 3W Dataset 2.0.0^21^. This diagram covers only the components necessary for a good understanding of how this dataset was conceived. In summary, it depicts the production platform, the well, the subsea Christmas tree^27^, the production and service lines, the electro-hydraulic umbilical, as well as sensors and valves.

The 3W Dataset 2.0.0^21^ contains 27 variables present in all instances. Following the adopted definition of MTS, each variable is included in an instance even if no data has been obtained or generated for that variable in a specific instance. Table 2 provides the name of each variable, its representation, and its physical position within the scenario depicted in Figure 1.

**Table 2:** Details of the variables in the 3W Dataset 2.0.0^21^.

Name	Description	Position
ABER-CKGL	Opening of the GLCK (gas lift choke)	1.1
ABER-CKP	Opening of the PCK (production choke)	2.1
ESTADO-DHSV	State of the DHSV (downhole safety valve)	3.1
ESTADO-M1	State of the PMV (production master valve)	4.1
ESTADO-M2	State of the AMV (annulus master valve)	5.1
ESTADO-PXO	State of the PXO (pig-crossover) valve	6.1
ESTADO-SDV-GL	State of the gas lift SDV (shutdown valve)	7.1
ESTADO-SDV-P	State of the production SDV	8.1
ESTADO-W1	State of the PWV (production wing valve)	9.1
ESTADO-W2	State of the AWV (annulus wing valve)	10.1
ESTADO-XO	State of the XO (crossover) valve	11.1
P-ANULAR	Pressure in the well annulus	12.1
P-JUS-BS	Downstream pressure of the SP (service pump)	13.1
P-JUS-CKGL	Downstream pressure of the GLCK	1.2
P-JUS-CKP	Downstream pressure of the PCK	2.2
P-MON-CKGL	Upstream pressure of the GLCK	1.3
P-MON-CKP	Upstream pressure of the PCK	2.3
P-MON-SDV-P	Upstream pressure of the production SDV	8.2
P-PDG	Downhole pressure at the PDG (permanent downhole gauge)	14.1
PT-P	Subsea Xmas-tree pressure downstream of the PWV in the production line	4.2
P-TPT	Subsea Xmas-tree pressure at the TPT (temperature and pressure transducer)	15.1
QBS	Flow rate at the SP	13.2
QGL	Gas lift flow rate	13.3
T-JUS-CKP	Downstream temperature of the PCK	2.4
T-MON-CKP	Upstream temperature of the PCK	2.5
T-PDG	Downhole temperature at the PDG	14.2
T-TPT	Subsea Xmas-tree temperature at the TPT	15.2

Note that some variable names contain terms or acronyms in Portuguese. For example: ABER = abertura = opening; CKGL = GLCK; CKP = PCK; ESTADO = state; ANULAR = annulus; JUS = jusante = downstream; and MON = montante = upstream. Translating all these names into English is a pending issue that will be resolved in future versions of 3W Dataset.

All instances were generated with observations recorded every 1 second, resulting in a fixed sampling frequency of 1 Hz.

All variables corresponding to a given physical quantity (type of variable) are expressed using the same measurement unit, as specified in Table 3.

**Table 3:** Physical quantities and their measurement units.

Physical Quantity	Measurement Unit
Choke opening	%
Flow rate	m^3^/s
Pressure	Pa
Temperature	°C
Valve state	Non-dimensional: 0 for closed, 1 for open, and 0.5 for any other state

The labeling process applied to all instances of the 3W Dataset 2.0.0^21^ resulted in two types of labels: class labels and state labels. Class labels are associated with normal conditions or the occurrence of undesirable events, while state labels are related to the operational status of the respective well.

The codes corresponding to the class labels are detailed in Table 4. Any of these codes can be assigned to any observation from any instance. Codes 101 to 109 represent transient conditions between normal operation and steady states associated with undesirable events. Therefore, there is no transient condition code associated with the class label Normal Operation. It is important to note that not all undesirable events have corresponding transient conditions. When the well condition in terms of normality or the occurrence of an undesirable event is unknown at any time, the associated observation is labeled with the class label Unknown (code = NaN = Not a Number). Additionally, each instance, as a whole, is associated with a single steady state code (from 1 to 9) that corresponds to at least part of its observations. This code is referred to as the type of event.

**Table 4:** Class labels and their codes.

Class Label	Steady State Code	Transient Condition Code
Normal Operation	0	—
Abrupt Increase of BSW	1	101
Spurious Closure of DHSV	2	102
Severe Slugging	3	—
Flow Instability	4	—
Rapid Productivity Loss	5	105
Quick Restriction in PCK	6	106
Scaling in PCK	7	107
Hydrate in Production Line	8	108
Hydrate in Service Line	9	109
Unknown	NaN	—

The following are succinct descriptions of each type of event:

- Abrupt Increase of BSW: a sudden rise in Basic Sediment and Water ratio, potentially leading to flow assurance issues, reduced oil production, and operational challenges;
- Spurious Closure of DHSV: an unexpected closure of Downhole Safety Valve, risking production losses;
- Severe Slugging: periodic and intense flow instability that can damage equipment and disrupt operations;
- Flow Instability: non-periodic flow disturbances that, if unaddressed, may escalate into severe slugging;
- Rapid Productivity Loss: a sudden decrease in well productivity, often driven by changes in system properties;
- Quick Restriction in PCK: a rapid and significant restriction in the production choke valve, typically caused by operational challenges;
- Scaling in PCK: formation of inorganic deposits in the production choke valve, reducing oil and gas production;
- Hydrate in Production Line: formation of crystalline compounds (hydrates) that can block pipelines, leading to significant production losses and high unblocking costs;
- Hydrate in Service Line: similar to Hydrate in Production Line, but occurring in the service line, leading to distinct signature patterns in operational data.
  For further details on these events, please refer to Vargas *et al*.^9^.
  The codes associated with the state labels are detailed in Table 5. These codes can be associated with any observation from any instance, but they are not associated with any instance as a whole. If the well’s operating condition is unknown at any given time, the respective observation is assigned the state label Unknown (code = NaN).

**Table 5:** State labels and their codes.

State Label	Code
Open	0
Shut-In	1
Flushing Diesel	2
Flushing Gas	3
Bullheading	4
Closed With Diesel	5
Closed With Gas	6
Restart	7
Depressurization	8
Unknown	NaN
  Each operational status is defined based on the configuration of the valves and the nature of recent and/or ongoing operations. In summary, the statuses are as follows:
- Open: all production valves (M1, W1, SDV-P, and PCK) are open, and auxiliary valves (PXO, and XO) are closed. The well is producing under regular condition;
- Shut-in: at least one valve in the production path is closed. The well is closed, so it is not producing;
- Flushing Diesel: at least one wellhead valve is closed, PXO or XO is open, and diesel is injected. The well is closed, and a diesel circulation operation from service line to production line is being executed;
- Flushing Gas: at least one wellhead valve is closed, PXO or XO is open, and gas is injected. The well is closed, and a gas circulation operation from service line to production line is being executed;
- Bullheading: all production valves are open, and diesel or gas is injected from the topside through the production line. The well is closed, and the production line is being pressurized from topside by diesel or gas to push down all the production fluids back into the well;
- Closed With Diesel: at least one production valve is closed, and the previous state was Flushing Diesel or Bullheading. The well is closed, and the majority of the production line is filled with diesel. This condition mitigates hydrates risk from occurrence;
- Closed With Gas: at least one production valve is closed, and the previous state was Flushing Gas. The well is closed, and the majority of the production line is filled with natural gas. This condition mitigates hydrates risk from occurrence;
- Restart: following a shut-in, all production valves are reopened. The well is recently opened, so it is in a transient period before it gets to the Open operational status;
- Depressurization: following a shut-in, SDV-P and PCK are open, while M1, W1, PXO, and XO remain closed. Production line is depressurized in order to mitigate hydrate risk from occurrence.
  Understanding and correctly identifying these operational statuses is critical in monitoring and preventing undesirable events. Some important additional explanations are as follows:
- Open state denotes continuous production with all key production path valves open and no auxiliary operations ongoing;
- All operational statuses other than Open are associated with well Shut-in procedures;
- Closed With Diesel state occurs following successful Flushing Diesel or Bullheading, during which the well is filled with diesel (a condition in which hydrate formation is highly unlikely);
- Bullheading is performed by injecting fluid (diesel or gas) directly from the topside into the production line under pressure, pushing the wellbore fluids back into the reservoir. This operation is used to ensure that a non-hydrate-forming fluid fills the system, often as a preventive action against hydrate plugs;
- Flushing Diesel, in contrast, is performed by circulating diesel through the service line and into the production flowline, displacing the original production fluids along the flowline path, typically toward the topside facilities. This operation ensures that hydrate-prone fluids are replaced with a more stable medium, reducing the chances of hydrate formation during shut-in;
- Closed With Gas state occurs following a Flushing Gas operation, which is performed by injecting and circulating gas (usually dry gas) via the service line into the production line. This operation also serves to remove liquid hydrocarbons or water that could contribute to hydrate formation, creating a drier environment inside the pipeline;
- Additionally, historical operational context can be valuable; for instance, depressurizing the production system is a typical measure performed shortly after the well is shut-in, in order to seek for thermodynamic conditions less favorable to the formation of hydrates;
- Depressurization state may indicate that subcooling levels were reduced, potentially mitigating hydrate risks during extended shut-ins.

### Method relating to real instances

The particularities of the method developed for real data are listed below.

1. Data acquisition:

1.1. Data is sourced from Petrobras’ PIMS^22^, specifically from different AVEVA PI System^23^ environments;
1.2. Linear interpolation provided by the AVEVA PI System is applied to simulate a fixed acquisition frequency of 1 Hz;
1.3. No preprocessing is performed for frozen variables or missing values;
1.4. Data is converted to standard measurement units.

**Fig. 2:**
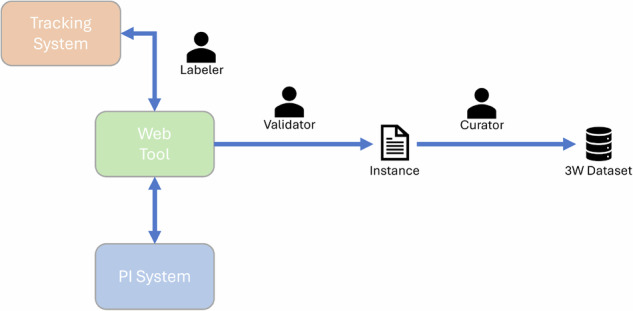
Illustration of the labeling process of real instances.
2.2. Historical records are mapped in a Tracking System to ensure accessibility and traceability for labelers (experts from Petrobras and its partners);
2.3. Labelers use a Petrobras’ Web Tool developed to label and export real data to the 3W Dataset;
2.4. An expert committee (Validators) reviews the labeling, suggests potential adjustments, and validates the assigned labels;
2.5. A Curator (a 3W Dataset specialist) finalizes the labeling process by updating the tool to mark validated events (instances) as labeled, enabling their exportation and inclusion in the 3W Dataset;
2.6. Figures 3 to 7 provide examples of labeled real instances. In these examples, only part of the variables is presented (before any standardization of measurement unit). These figures show the labels related to normal operation (light green), transient condition (yellow), and steady state (red) (Table 4) at the bottom of the figures. At the top of them, the state label (Table 5) is shown. In Figure 3, e.g., the top is dark green, meaning the well is open all the time.

2. Data labeling:

2.1. Figure 2 illustrates the steps of this process, which are described in the following items;

**Fig. 3:**
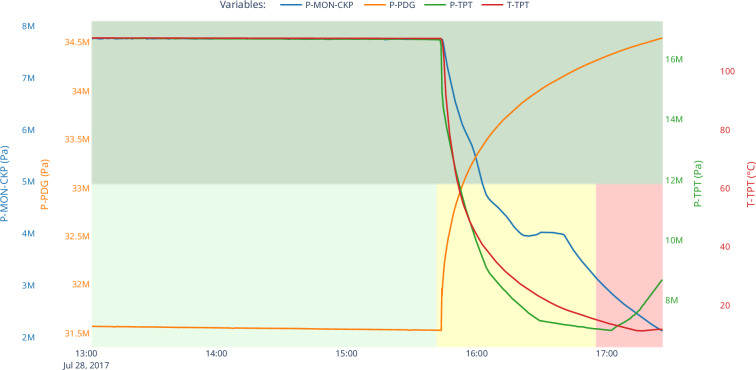
Example of a real Spurious Closure of DHSV instance in which pressure increase is observed in sensors located upstream of the DHSV (PDG) and pressure decreases are observed in sensors located downstream of the DHSV (TPT and MON-CKP). This example has 3 periods which were labeled with the Petrobras’ Web Tool as follows: light green (1st period = Normal Operation): class label = 0; yellow (2nd period = Transient Condition): class label = 102; red (3rd period = Steady State): class label = 2; dark green (Open): state label = 0.

**Fig. 4:**
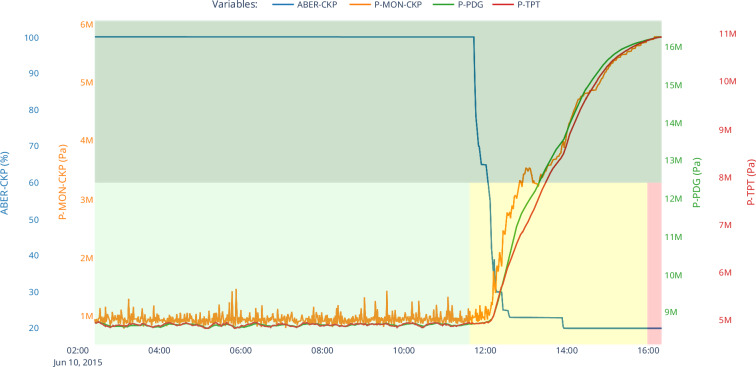
Example of a real Quick Restriction in PCK instance in which pressure increases are observed in sensors located in TPT, PDG, and MON-CKP. This example has 3 periods which were labeled with the Petrobras’ Web Tool as follows: light green (1st period = Normal Operation): class label = 0; yellow (2nd period = Transient Condition): class label = 106; red (3rd period = Steady State): class label = 6; dark green (Open): state label = 0.

**Fig. 5:**
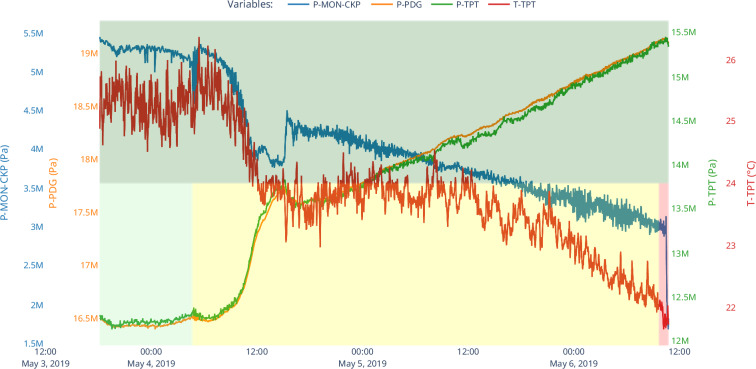
Example of a real Hydrate in Production Line instance in which pressure increases are observed in sensors located upstream of the production line (PDG and TPT) and pressure decrease is observed in sensor located downstream of the production line (MON-CKP). This example has 3 periods which were labeled with the Petrobras’ Web Tool as follows: light green (1st period = Normal Operation): class label = 0; yellow (2nd period = Transient Condition): class label = 108; red (3rd period = Steady State): class label = 8; dark green (Open): state label = 0.

**Fig. 6:**
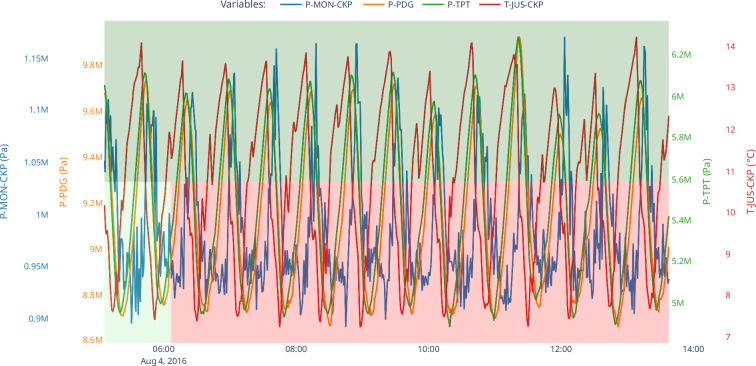
Example of a real Severe Slugging instance in which pressure oscillations with amplitudes above 10 bar are observed in sensors located in PDG and TPT. This example has 1 period which was labeled with the Petrobras’ Web Tool as follows: red (1st period = Steady State): class label = 3; dark green (Open): state label = 0.

**Fig. 7:**
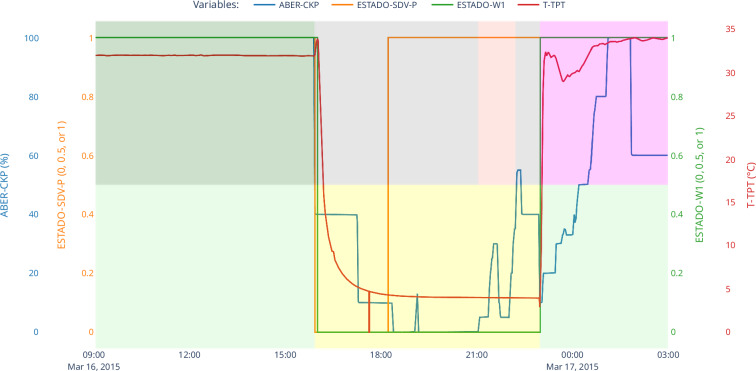
Example of a real Normal Operation instance in which depressurization has been carried out during a well shutdown, as shown by the opening of the PCK and SDV-P. This example has 1 period which was labeled with the Petrobras’ Web Tool as follows: light green (1st period = Normal Operation): class label = 0; dark green (Open): state label = 0; gray (Shut-In): state label = 1; salmon (Depressurization): state label = 8; magenta (Restart): state label = 7.

The main limitations of this method are:

1. It covers only events that occurred in real life and had archived records;
2. The mappings between variables and tags in PIMS were not verified. Therefore, data relating to certain variables may have been exported from PIMS and included in the 3W Dataset inappropriately. This is because it is known that variables in basically any PIMS may be temporarily associated with non-existent or outdated tags;
3. Original measurement units configured in PIMS (before conversions) were not verified.

### Method Relating to Simulated Instances

The particularities of the method developed for simulated data are listed below.

1. Data generation:

1.1. A matrix was designed for simulations with OLGA, gradually varying the main parameters of the considered scenario;
1.2. Simulations were run, and results that did not converge (e.g., wells that did not produce normally) were filtered out;
1.3. Time series were automatically extracted from TPL files generated by OLGA, corresponding to successful simulations;
1.4. Time series are perfectly periodic;
1.5. No frozen variables or missing values are present;
1.6. Variables are represented with standardized measurement units;
1.7. No noise is introduced in the time series.

2. Data labeling:

2.1. Labeling is fully automated based on simulation results, including transient condition periods.

The main limitations of this method are:

1. The method uses a single phenomenological model in OLGA associated with a single well;
2. A simplified simulation strategy was employed. For example: hydrate formation was simulated using a valve with a linear closing percentage;
3. Some variables were not included in the simulations, resulting in all their values being considered missing across all simulated instances.

### Method relating to hand-drawn instances

The particularities of the method developed for hand-drawn data are listed below.

**Fig. 8:**
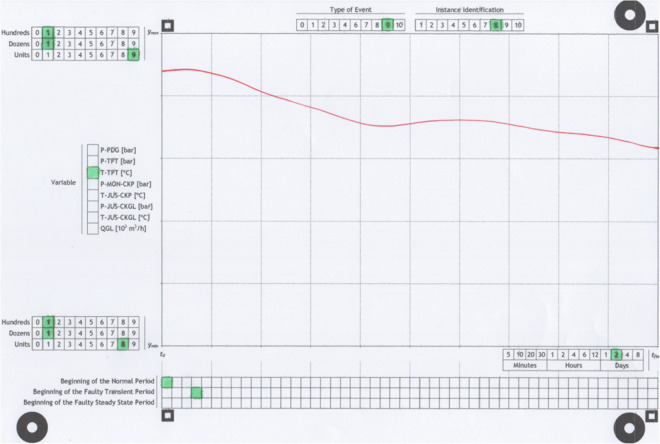
Example of a variable drawn and labeled by hand by an expert from Petrobras in the tool developed exclusively for generating instances for the 3W Dataset.

1. Data generation:

1.1. A proprietary tool based on image processing was developed exclusively for generating hand-drawn data for the 3W Dataset;
1.2. Each variable was hand-drawn on its own chart by an expert from Petrobras. An example is shown in Figure 8;
1.3. Time series were automatically digitized by scanning paper-printed graphs;
1.4. Time series are perfectly periodic;
1.5. No frozen variables or missing values are present;
1.6. Variables are represented with standardized measurement units.

2. Data labeling:

2.1. Labels were derived from expert markings on paper-printed graphs, including transient condition periods.

The main limitations of this method are:

1. The method covers only well-known signatures of the considered undesirable events;
2. There is potential for expert bias when drawing and labeling time series;
3. The process depends on the manual dexterity of the experts.

## Data Records

The 3W Dataset 2.0.0^21^ is licensed under CC BY 4.0^28^ and is publicly available at the following Figshare^29^ address: 10.6084/m9.figshare.29205836.v1.

In the root of the directory containing the dataset, there is a file called dataset.ini, which specifies properties of the 3W Dataset 2.0.0^21^. The proposal is that all users concentrate their searches for these properties in this file.

The data itself is organized into subdirectories dedicated to each type of event. The name of each directory corresponds to the code associated with each type of event (see Table 6).

**Table 6:** Quantities of instances that compose the 3W Dataset 2.0.0^21^.

Type of Event	Real	Simulated	Hand-Drawn	Total
0 - Normal Operation	594 (597)	0	0	**594 (597)**
1 - Abrupt Increase of BSW	4 (5)	114	10	**128 (129)**
2 - Spurious Closure of DHSV	22	16	0	**38**
3 - Severe Slugging	32	74	0	**106**
4 - Flow Instability	343 (344)	0	0	**343 (344)**
5 - Rapid Productivity Loss	11 (12)	439	0	**450 (451)**
6 - Quick Restriction in PCK	6	215	0	**221**
7 - Scaling in PCK	36 (4)	0	10	**46 (14)**
8 - Hydrate in Production Line	14 (3)	81	0	**95 (84)**
9 - Hydrate in Service Line	57 (0)	150 (0)	0	**207 (0)**
**Total**	**1119 (1025)**	**1089 (939)**	**20**	**2228 (1984)**

Values in brackets represent the quantities from version 1.0.0 that differ in the current version.

Each instance is stored in its own Apache Parquet file^30^, or simply Parquet file. Parquet is an open source, column-oriented data file format designed for efficient data storage and retrieval. It supports high-performance compression and encoding schemes, allowing it to handle complex data in bulk. It is also supported in many programming languages and analytics tools.

The logic used to formulate file names depends on the type of instance.

- Real Instances:
  - File names follow the format WELL-[incremental ID]_[timestamp of oldest observation].parquet. Example: WELL-00014_20170917140000.parquet;
  - Each real well is associated with a unique ID, regardless of the type of event (subdirectory). As each real well can give rise to one or multiple instances, the [timestamp of oldest observation] ensures unique identification.
- Simulated Instances:
  - File names follow the format SIMULATED_[incremental ID].parquet. Example: SIMULATED_00072.parquet;
  - The incremental ID starts at 1 for each type of event (subdirectory) and is sufficient to uniquely identify all simulated instances.
- Hand-Drawn Instances:
  - File names follow the format DRAWN_[incremental ID].parquet. Example: DRAWN_00007.parquet;
  - The incremental ID starts at 1 for each type of event (subdirectory) and is sufficient to uniquely identify all hand-drawn instances.

All Parquet files are created with the Pyarrow engine^31^ and the Brotli compression^32^. These choices provide a good compromise between compression ratio and read performance.

The timestamp vector of each instance is used as the index in the corresponding Parquet file. All timestamps are represented in the format ‘YYYY-MM-DD HH:MM:SS’.

All variables and labels are stored as columns in Parquet files, variables as float and labels as Int64 (not int64).

## Data Overview

The quantities of instances that compose the 3W Dataset 2.0.0^21^, categorized by type of instance and type of event, are presented in Table 6.

Types of events 0 and 4 are common, and we had no difficulty obtaining instances. On the other hand, types of events such as 1 and 6 are exceptional, and simulation was the option to provide instances of these types of events. Finally, the event Scaling in PCK is rare and requires a great deal of effort for simulation, justifying the manual generation alternative. In summary, the number of instances of each event type in the 3W Dataset is a result of these constraints.

**Fig. 9:**
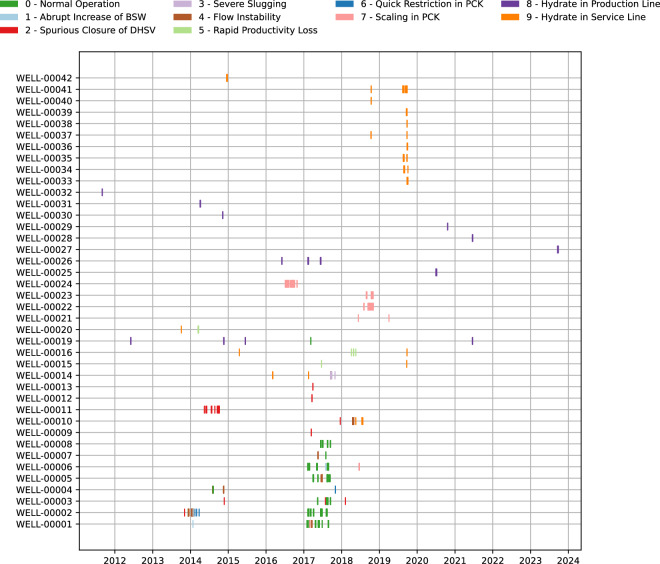
Scatter map with all the real instances in the 3W Dataset 2.0.0^21^.

Figure 9 shows a scatter map containing all the real instances. The oldest instance occurred in mid-2011, while the most recent occurred in mid-2023. In addition to the total number of wells considered (42), this map provides an overview of the distribution of instances over time and across wells.

The 3W Dataset’s main statistics related to inherent difficulties of real data are presented in Table 7.

**Table 7:** The 3W Dataset’s main statistics related to inherent difficulties of real data.

Statistic	Amount	Percentage
Missing Variables	41109	65.90% of 62384
Frozen Variables	6095	9.77% of 62384
Unlabeled Observations	4028400	5.26% of 76587318

### Main updates

The main updates introduced in version 2.0.0 compared to version 1.0.0 are summarized below. More details can be found in the release notes available in the 3W Project repository^17^.

1. The dataset structure has been significantly revised. Data is now stored in Parquet files instead of CSV files;
2. One variable (T-JUS-CKGL) has been discontinued, and 20 others have been added, resulting in 27 variables in the current version;
3. One new type of undesirable event has been added: Hydrate in Service Line;
4. The number of real instances has increased by 94;
5. The number of simulated instances has increased by 150;
6. The number of real wells covered has doubled, increasing from 21 to 42;
7. A new label, the state label, has been added;
8. More labeled observations have been incorporated across several instances;
9. No significant changes have been made to simulated or hand-drawn instances. All 20 new variables were incorporated into these instances with missing values.

## Technical Validation

Several carefully incorporated features in the methods described in the Methods Section ensure the high-technical quality of the 3W Dataset 2.0.0^21^. The most relevant features are the following:

- Real instances: preservation of real data characteristics, labeling by experts, and validation by expert committee;
- Simulated instances: simulation models calibrated by experts, and systematized labeling;
- Hand-drawn instances: hand-drawn graphs by experts, and systematized labeling.

The experts mentioned here are all from Petrobras and its partners.

## Usage Notes

This data article describes the 3W Dataset 2.0.0^21^, the current publicly available version, and summarizes its evolution from version 1.0.0, as previously detailed by Vargas *et al*.^9^.

The 3W Dataset 2.0.0^21^ was generated with Python 3.10^20^ code, using resources mainly provided by the Pandas 1.5^33^, and Pyarrow 19.0^31^ packages.

Designed for cross-platform compatibility, the 3W Dataset 2.0.0^21^ can be explored using virtually any programming language. However, regardless of the language, the Apache Parquet files^30^ must be accessed using Pyarrow engine^31^ alongside Brotli compression^32^.

When comparing results obtained in different works, it is important to distinguish which versions of the 3W Dataset were used. Due to substantial differences among the published versions, certain comparisons may pose challenges and require careful consideration.

The operational status of a well, represented by the state label, is strongly correlated with the values of its variables. This allows algorithms to be applied to the 3W Dataset for quantifying these relationships. Additionally, the state label can be used to select data for specific training purposes.

## Data Availability

The 3W Dataset 2.0.0^21^ is licensed under CC BY 4.0^28^ and is publicly available at the following Figshare^29^ address: 10.6084/m9.figshare.29205836.v1.

## Code availability

The 3W Project also develops and maintains the 3W Toolkit, a publicly available software package, hosted in its Git repository^17^. Written in Python 3^20^ and licensed under Apache 2.0^34^, this toolkit is designed to facilitate and encourage exploration of the 3W Dataset as well as the development and evaluation of comparative approaches. It provides a range of features for loading data, visualizing time series, calculating metrics, and more. Python practitioners can therefore use and adapt the 3W Toolkit to speed up their analysis with the 3W Dataset 2.0.0^21^.

An in-depth description of the 3W Toolkit is beyond the scope of this data article, but its documentation and examples of use can be found in the 3W Project repository^17^.
